# Presynaptic dopaminergic terminal imaging and non-motor symptoms assessment of Parkinson’s disease: evidence for dopaminergic basis?

**DOI:** 10.1038/s41531-016-0006-9

**Published:** 2017-01-25

**Authors:** MA Qamar, A Sauerbier, M Politis, H Carr, P A Loehrer, K Ray Chaudhuri

**Affiliations:** 1 National Parkinson’s Foundation International Center of Excellence, King’s College London and King’s College Hospital NHS Foundation Trust, London, UK; 2 Neurodegeneration Imaging Group, Department of Basic and Clinical Neuroscience, Institute of Psychiatry, Psychology and Neuroscience (IoPPN), King’s College London, London, UK; 3 Department of Neurology, University Hospital Cologne, Cologne, Germany

**Keywords:** Neurodegenerative diseases, Neurological disorders, Pathogenesis

## Abstract

Parkinson’s disease (PD) is now considered to be a multisystemic disorder consequent on multineuropeptide dysfunction including dopaminergic, serotonergic, cholinergic, and noradrenergic systems. This multipeptide dysfunction leads to expression of a range of non-motor symptoms now known to be integral to the concept of PD and preceding the diagnosis of motor PD. Some non-motor symptoms in PD may have a dopaminergic basis and in this review, we investigate the evidence for this based on imaging techniques using dopamine-based radioligands. To discuss non-motor symptoms we follow the classification as outlined by the validated PD non-motor symptoms scale.

## Introduction

Contrary to previous perceptions, Parkinson’s disease (PD) is recognised as a multisystem disorder. Besides dopamine (DA), three further key neurotransmitters have been described to be involved in the pathogenesis of PD; namely noradrenaline (NA), acetylcholine (ACh), and serotonin (5HT).^1,2^ Consequentially, non-motor symptoms (NMS) in PD can potentially be related to dopaminergic, non-dopaminergic pathogenesis or a combination of both.^1,3^ Individual studies indicate that apathy,^4^ anxiety^5^ as well as aspects of sleep disturbances^6^ appear to be linked to striatal dopaminergic deficiency as measured by dopamine transporters (DaT) scans. However, NMS such as depression,^7^ fatigue,^8^ weight changes,^9^ and visual hallucinations (VH)^10^ may be driven by deficiency in non-dopaminergic transmitters.

The NMS Scale (NMSS) was validated as the first comprehensive and holistic health-professional completed measure of NMS in PD and has now been used as a primary or secondary outcome measure in a number of clinical trials and epidemiological studies.^11^ The NMSS allows for calculation and grading of the burden (severity multiplied by frequency) of 30 different NMS, which are covered in nine different domains.^12,13^ These are cardiovascular, sleep/fatigue, mood/cognition, perceptual problems/hallucinations, attention/memory, the gastrointestinal tract, urinary system, sexual function, and miscellaneous containing olfactory dysfunction.

**Fig. 1:**
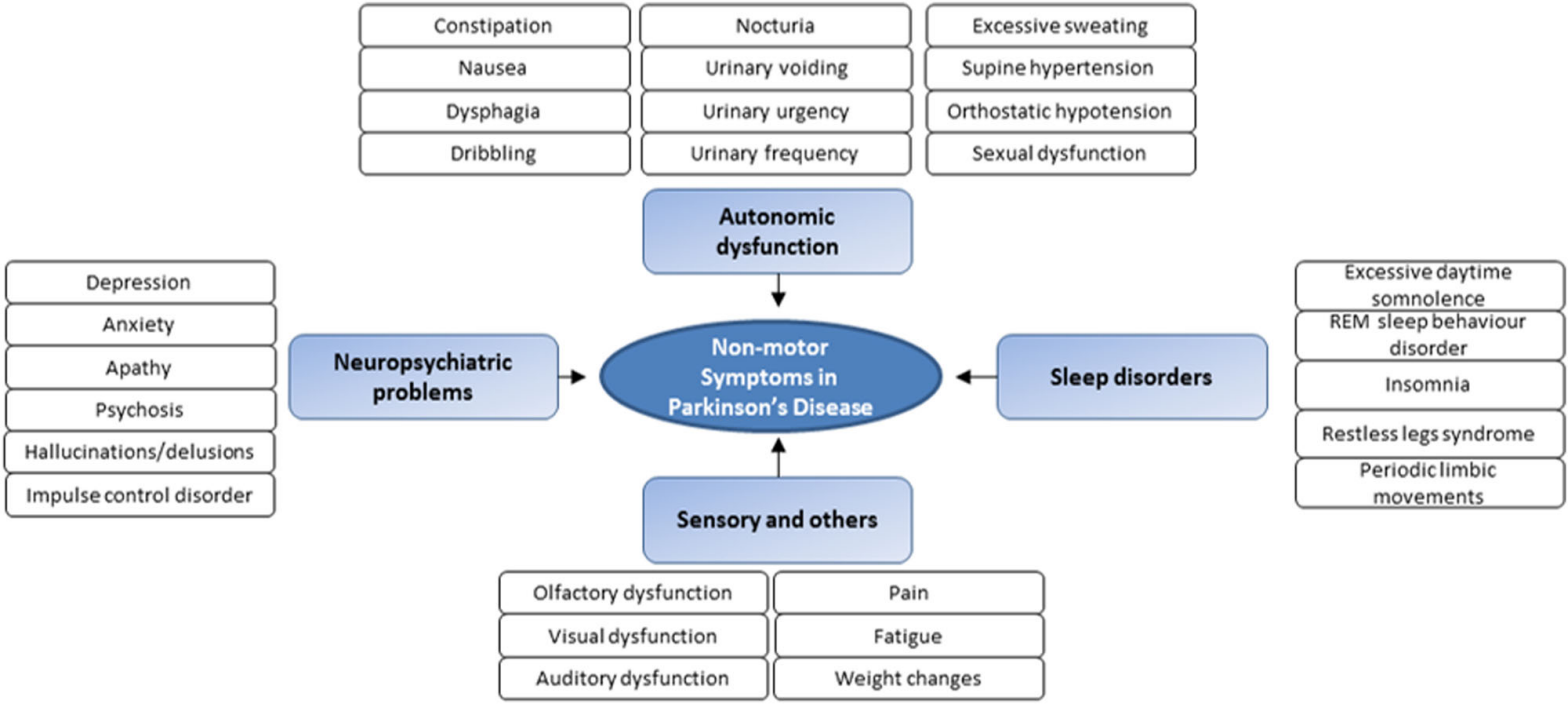
Diagrammatic representation of the common NMS in PD, as included in the non-motor symptoms scale (NMSS). *REM* rapid eye movement

In this review we primarily address the relationship of dopaminergic radioligands and the individual NMS covered by NMSS domains to examine a possible underlying dopaminergic basis of these varying NMS (Fig. 1).

**Fig. 2:**
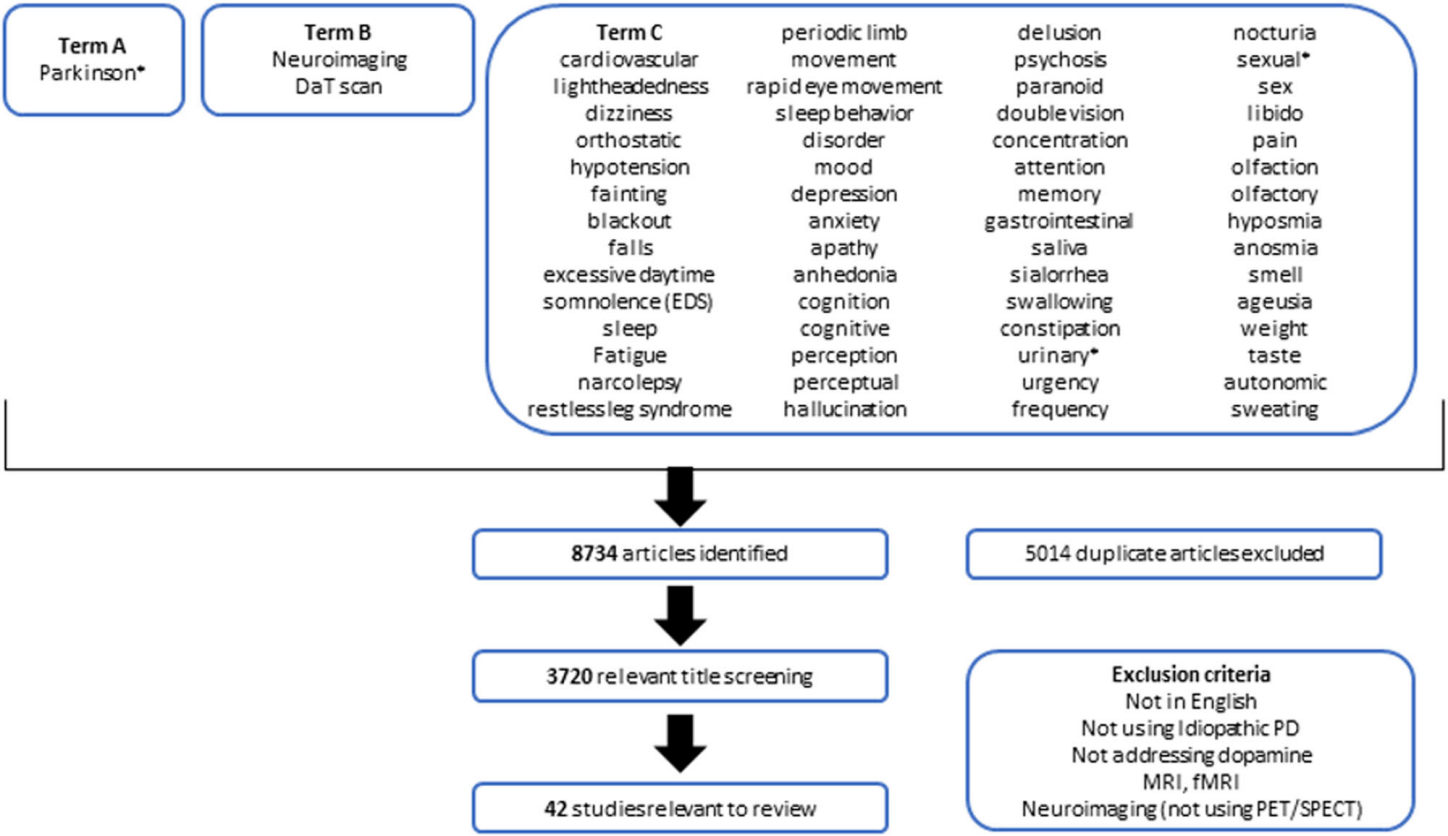
Methodology used for this review. *DaT* dopamine transporter, *EDS* excessive daytime somnolence, *MRI* magnetic resonance imaging, *fMRI* functional MRI, *PET* position emission tomography, *SPECT* single positron emission computed tomography, *PD* Parkinson’s disease

To conduct this review, we gathered articles, which used dopaminergic imaging to explore the pathophysiology of different NMS, Fig. 2 summarises our methodology. We had three possible terms; Term A had an asterisk allowing for several terms with the same beginning being considered. Term B covered neuroimaging words, whilst Term C were the possible NMS that could have been used. We initially found 8734 articles, which then left us with 42 studies to include once we removed duplicated and referred to our exclusion criteria.

## NMSS domain 1: cardiovascular dysfunction

Cardiovascular dysfunction is a key autonomic feature of PD and patients often present with orthostatic hypotension (OH). There are currently no studies reporting cardiovascular dysfunction in PD to have a dopaminergic basis. However, using the ^123^I-meta-iodobenzyl guanidine (MIBG) radiotracer, a NA analogue, studies have shown there to be a reduction in the postganglionic presynaptic cardiac sympathetic innervation, suggestive of cardiac sympathetic dysfunction early in PD patients, giving rise to symptoms such as OH.^14–19^

Several studies have shown reduction in cardiac uptake regardless of using MIBG or ^18^F-DOPA cardiac positron emission tomography (PET) in PD patients with OH when compared to those without.^14–16,20,21^ These results suggest that there might be a decrease in catecholamine uptake (that being DA or NA) in PD patients with OH, but not all studies agree.^22,23^

### NMSS cardiovascular dysfunction: summary statement

There is evidence of sympathetic neuronal defect, specifically focusing on noradrenergic depletion in PD.^23–27^ However, there are no dopaminergic imaging studies exploring a dopaminergic defect as the basis of cardiovascular dysfunction. In line with the assumption of initial lower brainstem involvement,^28^ noradrenergic dysfunction likely occurs prior to dopaminergic dysfunction, prompting suggestions that PD may be partly a noradrenergic disorder.^29^ Further research is needed to explore dopaminergic involvement and noradrenergic dysfunction in early PD patients.

## NMSS domain 2: sleep disorders and fatigue

Sleep disturbance occurs in 60–90% of all PD patients^30^ with symptoms ranging from insomnia, sleep apnoea, restless legs syndrome (RLS), rapid eye movement (REM) behaviour disorder (RBD) to excessive daytime somnolence (EDS). It represents one of the most frequent complaints by the patients.^31^ Two aspects of sleep dysfunction, EDS and RBD, have been studied with dopaminergic imaging and are discussed below.

- Excessive daytime somnolence

EDS is the tendency to drift off to sleep quickly and more frequently than usual during the day.^32^ EDS can be assessed by the Epworth sleepiness scale (ESS).^33^ The link between EDS and dopaminergic dysfunction is unclear. Pavese and colleagues conducted a multi-modal PET study, using ^18^F-DOPA and ^11^C-DASB tracers in PD patients with EDS, and reported both dopaminergic and serotonergic dysfunction (Table 1).^34^ Happe and colleagues had previously proposed dopaminergic dysfunction underlying EDS in PD and additionally, Pavese and colleagues used C-DASB, a serotonin transporter binding ligand, and showed evidence of serotonergic dysfunction in the raphe area of the brain in PD. However, a recent study by Qamhawi and colleagues reported no significant correlation between raphe binding with ^123^I-FP-CIT-SPECT and EDS.^35^

**Table 1:** Dopaminergic basis of NMSS Domain 2 (Sleep and Fatigue) pathophysiology in PD

Author	Year	NMS	Radiotracer	Demographics	Results	Analysis
Happe et al.^203^	2007	EDS	^123^I-FP-CIT SPECT	21 PD patients (14 de novo, 7 pre-levodopa treated). Examination was via imaging, H&Y, UPDRSIII, ESS, PDSS, SDS	Significant negative correlation of ESS and mean DaT binding on both sides of striatum (*r* = −0.63, *p* = 0.03); putamen (*r* = −0.60, *p* = 0.04); caudate (*r* = −0.71, *p* = 0.01).	The study suggests daytime sleepiness to have a dopaminergic nigrostriatal defect. Using de novo PD patients, which is its strength in this clinical study however, there were no controls used. Surprisingly, patients with H&Y two contralateral vs. ipsilateral showed no significant difference in DaT binding. Having a low sample size would potentially explain why no correlation was observed between nigrostriatal DaT binding with duration and severity of the disease.
Eisensehr et al.^43^	2000	RBD	^123^I-IBZM, ^123^I-IPT	5 RBD, 14 PD patients, 7 controls.	Significantly reduced ^123^I-IPT binding compared to controls (*p* = 0.003). Contralateral striatum was significantly higher in ^123^I-IPT binding compared to symptomatic PD patients (*p* = 0.02). ^123^I-IBZM did not produce any significant difference between RBD and controls.	The results suggest a reduced striatal DaT to be found in iRBD. This is a controlled study, one of the earliest to address RBD, therefore an important study. However the low sample size makes comparison difficult.
Eisensehret al.^44^	2003	RBD	^123^I-IBZM, ^123^I-IPT	16 iRBD, 8 PD patients (H&Y S1) and 11 controls.	Significant decrease in ^123^I-IPT uptake in iRBD patients from controls (*p* = 0.001), but ^123^I-IBZM uptake was not significantly different between any groups.	This is a controlled study underpinning a dopaminergic dysfunction in RBD.
Schifitto et al.^62^	2008	Fatigue	^123^I-β-CIT	361 PD patients enroled in a randomised, double-blind, placebo-controlled ELLDOPA trial	Fatigue-PD patients had least uptake in the putamen (*n* = 49, m = 2.65, SD = 1.61), which is also the case for non-fatigue-PD (*n* = 82, m = 2.42, SD1.09).	There were no significant different in ^123^I-β-CIT uptake between fatigue and non-fatigue PD suggesting alternative non-dopaminergic pathways, such as noradrenergic dysfunction underpinning the pathophysiology of fatigue.
Kim et al.^204^	2010	RBD	^123^I-FP-CIT	14 RBD and 14 PD patients and 12 controls underwent imaging and EMG analysis	RBD patients had a significantly higher DaT binding in the striatum than PD patients. DaT binding was significantly lower compared to controls in the putamen only (*p* = 0.02) but not the collective striatum (*p* = 0.07). DaT density in the putamen in early-PD was below normal ranges.	The study concludes that the dopaminergic system is involved but may not be essential for RBD development. This is a controlled study making its results important and validated; the diagnosis of RBD would be interesting to know but is not mentioned.
Pavese et al.^8^	2010	Fatigue	^18^F-DOPA, ^11^C-DASB	10 non-fatigue-PD and 10 fatigue-PD patients enroled	Fatigue patients had significantly lower SERT binding than patients without fatigue in the caudate, putamen, ventral striatum and thalamus (*p* = 0.01). Striatal ^18^F-dopa uptake was similar in the fatigued and non-fatigued groups, however there was a trend towards a lower mean uptake in fatigue-PD (*p* = 0.095).	Fatigue seems to have more of a serotoninergic dysfunction than dopaminergic. This supports the notion of non-motor subtyping as a possible biomarker for Park fatigue.
Pavese et al.^34^	2012	EDS	^18^F-DOPA, ^11^C-DASB	11 PD patients with EDS, 10 PD patients without EDS.	PD-EDS had significant decrease in SERT binding in the thalamus (*p* < 0.001), locus coeruleus (*p* < 0.001), rostral raphe (*p* < 0.05), hypothalamus (*p* < 0.05). There was significant reduction in ^18^F-DOPA uptake in the locus coeruleus (*p* < 0.001), rostral raphe (*p* < 0.05), and VTA (*p* < 0.05).	A monoaminergic dysfunction is proposed by this study, particularly limbic serotonergic functions. The study highlights non motor subtyping, particularly Park-sleep phenotype. Controlling for depression and fatigue is a strength of this study.
Moccia et al.^6^	2016	RLS	^123^I-FP-CIT	109 newly diagnosed drug-naïve PD patients underwent 2- 4-year follow-up for RLS.	By using DaT scan at baseline to access DaT availability, they have found an increase in DaT availability in caudate and putamen to be more likely associated with baseline RLS (*n* = 5, OR = 75.7, *p* = 0.077) and RLS follow-up (*n* = 16, OR = 12.0, *P* = 0.059).	This is very important data in relation to controversial concepts of RLS in PD. The study suggests that PD patients with RLS have comparatively preserved dopaminergic pathways.

^*11*^ *C-DASB* [11C]-labelled 3-amino-4-(2-dimethylaminomethyl-phenylsulfanyl) benzonitrile, ^*123*^ *I-CIT* [(123)I]2beta-carbomethoxy-3-(4-iodophenyl)tropane, ^*123*^ *I-FP-CIT* [123I]-2β-carbomethoxy-3β-(4-iodophenyl)-N-(3-fluoropropyl) nortropane, ^*123*^ *I-IBZM* [123I]Iodobenzamide, ^*123*^ *I-IPT* I-123N-(3-iodopropen-2-yl)-2beta-carbomethoxy-3beta-(4-chlorophenyl)tropane, ^*18*^ *F-DOPA* 18F-dihydroxyphenylalanine, *DaT* dopamine transporter, *EDS* excessive daytime sleepiness, *EMG* electromyography, *ESS* Epworth Sleepiness Scale, *H&Y S* Hoehn and Yahr scale stage, *iRBD* idiopathic RBD, *PET* positron emission tomography, *PD* Parkinson’s disease, *PDSS* Parkinson’s disease sleep scale, *RBD* rapid eye-movement behavior disorder, *RLS* restless leg syndrome, *SDS* self-rating depression scale, *SERT* serotonin transporter, *SPECT* single photon emission tomography, *UPDRSIII* Unified Parkinson’s disease rating scale motor score.

- Rapid eye movement sleep Behaviour Disorder (RBD)

REM sleep behaviour disorder (RBD) is characterised by the loss of muscle inhibition during REM sleep, which leads to the physical acting out of violent and dangerous nightmares.^36,37^ RBD can entirely be an idiopathic disease (iRBD) or secondary to neurodegenerative conditions such as multiple system atrophy (MSA) or Dementia with Lewy Bodies (DLB). In the field of PD, 60% of PD patients experience RBD^38^ and 80% of iRBD patients progressing to PD in 10–12 years.^3^ Hence, RBD is now recognised as the most robust marker of prodromal PD.^39^ RBD has been shown, at least in part, to be associated with dopaminergic defect, which is consistent with Braak stage 2 pathophysiology.^28^ Reduction in DaT uptake in iRBD patients has been shown, specifically in the putamen.^40,41^ Using ^123^I-FP-CIT, ^123^I-IBZM, and ^11^C-dihydrotetrabenazine (DTBZ) radiotracers, several studies have suggested the nigrostriatal dopaminergic pathway as being implicated in RBD pathogenesis.^42–45^ However, Kim and colleagues reported their idiopathic RBD patients had a reduced DaT uptake in the putamen, yet when assessing DaT density in the putamen, the levels remained within normal ranges, leaving them to conclude there may likely be an additional pathogenic pathway implicated in RBD (see Table 1). Studies exploring REM sleep duration, using PET and SPECT imaging, have yielding interesting results whereby the upper brainstem is found to be suppressing REM sleep in early-PD causing uncertainty as to a dopaminergic or non-dopaminergic involvement in the pathophysiology of REM-sleep in PD itself.^46,47^ Investigating non-dopaminergic nuclei has led to assumptions of RBD pathophysiology to include the pedunculopontine nucleus and laterodorsal tegmental nuclei (cholinergic nuclei), raphe nucleus (serotoninergic), pre-coeruleus (glutaminergic) and locus coeruleus (noradrenergic).^48,49^

- Restless Leg Syndrome (RLS) and Periodic Limb Movements (PLM)

RLS and PLM are common in PD patients.^50^ The precise pathophysiology of both conditions is still unknown and, to our knowledge, there are currently no specific studies investigating RLS-PD pathophysiology using dopaminergic imaging. Nonetheless, studies in idiopathic RLS have suggested a dopaminergic mechanism central to its pathophysiology, which is made evident by the effectiveness of dopaminergic treatment.^51^ Studies have shown there to be hypo-dopaminergic activity in idiopathic RLS patients either through reduction in DaT uptake, densities or receptor availability.^52–56^ However, others have shown no such change,^57–59^ while some have even reported an increase in DaT densities.^60^ Furthermore, it has been hypothesised that compared to PD patients, idiopathic RLS patients may have a mishandling of DA rather than a decrease of dopaminergic cells, as seen in PD.

- Fatigue

Fatigue is a specific NMS in PD with considerable negative impact on the quality of life of patients.^61^ Some studies reported fatigue and dopaminergic dysfunction as not being significantly associated when assessed using neuroimaging.^8,62,63^ Both, Schifitto and colleagues and Pavese and colleagues found no significant reduction in striatal dopaminergic uptake between fatigued and non-fatigued PD patients (Schifitto et al. 2008, Pavese et al. 2010). However, Pavese also used ^11^C-DASB PET and reported a significant reduction in serotonergic transporter (SERT) binding particularly in the caudate, putamen, ventral striatum and thalamus (Table 1). Hence, Pavese suggests, not only the involvement of extra-striatal pathways, but also a non-dopaminergic involvement in the form of serotonergic dysfunction underpinning central fatigue.

Supplementary to the notion of a non-dopaminergic involvement, Chou and colleagues recently hypothesised cholinergic dysfunction to also be involved by using ^11^C-methyl-4-piperidinyl propionate (PMP) acetylcholinesterase (AChE) and ^11^C-DTBZ monoaminergic PET imaging.^63^ However, their results found no significant evidence to support this hypothesis. Nonetheless, clinical experience dictates that dopaminergic therapies can be effective in treating fatigue-PD patient groups, which has led many to conclude that dopaminergic dysfunction might have a partial role.^62,63^

### NMSS sleep disorders and fatigue: summary statement

Sleep disorders in PD, particularly EDS and RBD, may both have a dopaminergic basis, at least in part. Complex pathway interactions underpin RBD where cholinergic mechanisms are also implicated, while raphe serotonergic dysfunction may underlie EDS. DaTscan imaging, such as using ^18^F-DOPA, has provided little evidence to support a dopaminergic basis to fatigue in PD, instead a non-dopaminergic pathway (such as limbic serotonergic deficit) seems more plausible.

## NMSS domain 3: mood and apathy

Neuropsychiatric problems are a common manifestation in PD^64^ with depression being the most prevalent with up to 45% of patients affected.^65^ Here, we explore the most commonly discussed mood and apathetic problems PD patients face and examine its potential pathophysiology using different radiotracers with a focus on DA.

- Depression

The pathophysiology of depression has been associated with dopaminergic defect by several studies (Table 2) reporting an inverse correlation of depression with dopaminergic availability.^66–70^ However, as Braak hypothesis suggests, early lower brainstem pathophysiology may involve several other nuclei and thus other neurotransmitters,^28^ specifically serotonin.^71^ When investigating this association, studies have found there to be an inverse correlation between SERT binding within areas such as the dorsal midbrain, suggestive of a serotonergic dysfunction.^72,73^ Politis and colleagues conducted a large in vivo study, using ^11^C-DASB PET in antidepressant-naïve PD patients. Their results show an altered serotonergic function associated with higher depression levels in these patients, which suggests abnormal serotonergic neurotransmission in PD depression pathophysiology. Further probing into alternative neurotransmitter involvement has found there to also be noradrenergic dysfunction in the locus coeruleus.^69,74^

**Table 2:** Dopaminergic basis of NMSS Domain 3 (mood and apathy) pathophysiology in PD

Author	Year	NMS	Radiotracer	Demographics	Results	Analysis
Remy et al.^69^	2005	Depression	^11^C-RTI32	20 PD patients (dPD, *n* = 8; ndPD, *n* = 12). dPD diagnosis was made using DSM-IV criteria.	The bilateral locus coeruleus, bilateral dorsomedial and inferior thalamui, left ventral striatum, and right amygdala had a significant reduction (*P* < 0.01) of ^11^C-RTI32 binding in the depressed compared to non-depressed PD patients.	There seems to be both dopaminergic and noradrenergic defect in the limbic system of dPD, as suggested by the results. The study’s sample is small and their PD patient’s disease duration ranges from 0.5 to 9 years, which is very broad. However, this is an important study and a first of its kind, underpinning noradrenergic as well as dopaminergic dysfunction in anxiety and depression.
Weintraub et al.^66^	2005	Depression	^99m^Tc-TRODAT-1	76 PD patients and 46 healthy controls underwent SPECT with ROIs calculated from 6 regions.	A significantly lower DaT uptake was noted in all regions of PD patients (all ROIs, *P* < 0.001). Left anterior putamen DaT availability (*r* = −0.24, *p* = 0.05) was most significant.	The author’s findings suggest striatal dopaminergic dysfunction is likely necessary for the development of affective symptoms, such as depression, in PD. A robust sample size for an imaging study, but low DaT uptake is non-specific and has been linked to many NMS and motor syndromes of PD.
Koerts et al.^205^	2007	Depression	^18^F-DOPA	23 PD patients assessed using MADRS to not have depression underwent PET.	MADRS total correlated with mean dopaminergic activity in bilateral putamen (*r* = −0.44, *p* = 0.02) and caudate (*r* = −0.50, *p* = 0.01).	The study results suggest striatal dopaminergic dysfunction pathophysiology in dPD. However, they use the MADRS, a cognitive assessment arm of a depression scale; hence there suggestion of depression to be dopaminergic in basis is confusing as they are only assessing the cognitive aspect. Furthermore, they used a one-tailed correlation between MADRS and mean FDOPA. The lack of a control group is a problem.
Rektorova et al.^67^	2008	Depression	^123^I-FP-CIT	20 PD patients with and 20 patients without depressive symptoms and cognitive impairment were assessed using TOL and MADRS against their DaT uptake in various regions.	Hypo-dopaminergic function in the left striatum (*r* = −0.52, *p* = 0.018) and left putamen (*r* = −0.55, *p* = 0.012) recorded in PD patients. Multiple linear regression analysis supports a strong dopaminergic association between MADRS score and DAT uptake in the left striatum (*p* = 0.005) and left putamen (*p* = 0.003).	Dopaminergic defect very likely exists in dPD, is concluded in the study. This is a comparative study but no control group. The association of left sided ^123^I-FP-CIT uptake is of interest.
Hesse et al.^68^	2009	Depression	^123^I-FP-CIT	140 PD patients (dPD, *n* = 30; ndPD, *n* = 110) had their striatum, thalamus and midbrain/brainstem regions imaged using SPECT. Depression was a subjective of symptoms present and SCID. 13 patients were on SSRIs. 18 suitable controls were included.	dPD had a significantly lower uptake in the striatum (*p* < 0.001), thalamus (*p* = 0.002), and midbrain/brainstem (*p* = 0.025).	The study concludes dPD had loss of striatal DaT availability caused by dopaminergic dysfunction and dopaminergic neuronal loss. This large study however is not properly controlled. The outcome is not surprising and the conclusions are rather complex.
Felicio et al.^210^	2010	Depression	^99m^Tc-TRODAT-1	10 ndPD patients and 10 dPD patients were assessed with SPECT and BDI score.	dPD patients had higher DaT density in left caudate (*p* = 0.02) and right putamen (*p* = 0.03) than ndPD patients.	Since DaT density increases in Dpd, they suggest a DaT pathophysiology may likely be at play. But the use of ^99m^Tc-TRODAT-1 for a small study makes it difficult to come to any definitive conclusion.
Di Giuda et al.^70^	2012	Depression	^123^I-FP-CIT	21 PD patients had the HDRS, HARS, SHPS performed to assess anxiety and depression.	A strongly significant inverse correlation was found between severity of depression symptoms and DaT availability in the left caudate (*r* = −0.63, *p* = 0.002).	Dopaminergic dysfunction could be the pathologically relevant in dPD. The study doesn’t allow for additional assessments due to the cross-sectional design of the study, which may obscure an accurate psychiatric diagnosis. However, the study points towards a role of the caudate in neuropsychiatric and other NMS of PD. The relation with left caudate is of interest.
Ceravolo et al.^206^	2013	Depression	^123^I-FP-CIT	44 PD patients assessed using HAM-D and BDI and underwent SPECT imaging.	Bilateral striatal DaT uptake was positively correlated with both HAM-D (*r* = 0.329; *r* = 0.423, right and left respectively) and BDI (*r* = 0.377; *r* = 0.360, right and left respectively) in dPD, after controlling for confounders (*p* < 0.05).	The study data is consistent with previous evidence that affective symptoms are correlated with increased DaT density.
Vriend et al.^207^	2014	Depression	^123^I-FP-CIT	100 non-demented PD patients underwent assessment using BDI and SPECT.	Severity of depression had an inverse correlation with DaT binding in the right caudate (*r* = −0.27, *p* = 0.007), however no significant difference was observed elsewhere. UPDRS-III score was significantly associated with DaT binding ratio in the right putamen (*β* = −0.26, *p* = 0.03) but not in the right caudate (*β* = −0.09, *p* = 0.38).	Depressed PD may be associated with DA deficit in the caudate nucleus, whilst motor symptoms accrue in part from putaminal dopaminergic deficit. This is an important study suggesting differential motor and non-motor roles of putamen and caudate in PD. However, there was no clinical diagnosis of depression which was discussed in the study, nonetheless the use of a robust sample size plays favourably for the study.
Kaasinen et al.^208^	2001	Anxiety	^18^F-DOPA	47 PD patients underwent PET and MRI. All completed the TCI and KSP for personality trait diagnosis	Personality traits in PD and anxiety (somatic or psychic) had a positive correlation with DAT uptake in the caudate (*r* = 0.39 to 0.49, *p* < 0.01) however, statistical significance was lost after correction for cofounders.	Interesting work although the data is insufficient to produce any specific conclusions.
Remy et al.^69^	2005	Anxiety	^11^C-RTI32	20 PD patients were diagnosed. Anxiety was measured using State Trait Anxiety inventory.	Anxiety score was negatively correlated with binding potential values in left ventral striatum, left caudate, left locus coeruleus, left inferior thalamus and bilateral amygdala and medial thalamus (*p* = 0.05).	Inverse relationship between the binding of [11C]-RTI32 in these regions and the severity of anxiety and mood disorders in these patients suggests a potential for both a dopaminergic and noradrenergic basis. This is an important PET study addressing a multi-neuro-transmitter basis of anxiety and depression in PD.
Weintraub et al.^66^	2005	Anxiety	^99m^Tc-TRODAT-1	76 PD patients and 46 healthy volunteers were assessed using the STAI and POMS.	PD patients showed there to be a negative correlation using both State and Trait anxiety parameters with DaT uptake in the right anterior putamen (state anxiety [*r* = −0.24, *p* = 0.04], Trait anxiety [*r* = −0.30, *p* = 0.01]).	Controlled data and this data is consistent with previous work in that dopaminergic dysfunction may be necessary for affective symptom development.
Moriyama et al.^78^	2011	Anxiety	^99m^Tc-TRODAT-1	32 PD patients who were assessed and diagnosed as having generalised SAD (*n* = 11) according to DSM-IV criteria.	A positive correlation, using the Brief Social Phobia Scale (BSPS), was found specifically in the right (*r* = 0.37, *p* = 0.04), left putamen (*r* = 0.43, *p* = 0.02), and left caudate (*r* = 0.39, *p* = 0.03).	The study suggests a dopaminergic defect is plausible within the pathophysiological realms of social anxiety PD. However, another study suggests dopaminergic basis of anxiety.
Di Giuda et al.^70^	2012	Anxiety	^123^I-FP-CIT	21 PD patients had the HDRS, HARS, SHPS performed to assess anxiety and depression.	Using HARS cut/off of 10/11 there was no significant difference in DaT availability between anxiety-PD patients (*n* = 17) and those without anxiety (*n* = 4), but this showed a trend towards lower uptake in the left caudate (*p* = 0.07) of anxious-PD patients.	The study used a very small sample size which makes any meaningful comparison between anxious vs. non-anxious patients difficult.
Erro et al.^5^	2012	Anxiety	^123^I-FP-CIT	34 untreated PD patients evaluated using HADS-D, HADS-A scales, and BDI.	Inverse correlation between the severity of anxiety and nigrostriatal DaT availability within the right caudate (*r* = −0.39, *p* = 0.01) and left caudate (*r* = −0.31, *p* = 0.03).	A potential association between DaT defect and anxiety-PD symptoms as noted before. The untreated PD cohort is strength of this study.
Remy et al.^69^	2005	Apathy	^11^C-RTI-32	20 PD patients had apathy measured using the AES and STAI.	Negative correlation with apathy score and ^11^C-RTI-32 binding potential values in the left ventral striatum, left caudate and left coeruleus, left inferior thalamic region and bilateral amygdala and medial thalamus.	The use of ^11^C-RTI-32 as both a dopaminergic and noradrenergic marker is interesting. As these patients were also assessed for depression (see above), the study suggests that depression and anxiety in PD is correlated with both loss of noradrenergic and dopaminergic pathways in the limbic system.
Santangelo et al.^4^	2015	Apathy	^123^I-FP-CIT	14 PD patients with pure apathy and 14 PD patients without, underwent AES-S and imaging.	Results showed low DaT levels in the striatum, with only the right caudate (*p* = 0.006) being significant in apathetic PD patients.	The study design used patients medicated at time of apathy assessments, hence the influence of dopaminergic medication cannot be ruled out and patients with mild cognitive impairments may have been included in the analysis. However, the study underpins the dopaminergic dysfunction basis of apathy. Comparative and therefore of potential use, however, contrary to data showed by Chaung et al 2016.
Chung et al.^88^	2016	Apathy	^18^F-FP-CIT	20 pure apathy PD patients assessed using AES-S and scans	Results show pure apathy PD patients show no statistically significant difference of striatal DaT compared with non-apathetic patients. The right anterior putamen (*r* = 0.064, *p* = 0.516) and right posterior putamen (*r* = 0.124, *p* = 0.117).	The study concludes that dopaminergic depletion of the striatum does not correlate with apathy in early PD. The results here are contradictory to that of Santangelo et al 2015. Furthermore, the smaple size is very small and there is no comparative group.

^99m^ *Tc-TRODAT-1* technetium-99m [2-[[2-[[[3-(4-chlorophenyl)-8-methyl-8-azabicyclo[3,2,1]oct-2-yl]methyl] (2-mercaptoethyl) amino]ethyl] amino] ethanethiolato(3-)-N2,N2’,S2,S2’]oxo-[1R-(exo-exo)], ^11^ *C-RTI32* [11C](–)-2β-Carbomethoxy-3β-(4-tolyl)tropane, ^123^ *I-FP-CIT* [123I]-2β-carbomethoxy-3β-(4-iodophenyl)-N-(3-fluoropropyl) nortropane, ^18^ *F-DOPA* 18F-dihydroxyphenylalanine, *AES-S* apathy evaluation scale, *BDI* beck depresion inventor, *BR* dopamine transporter binding ratio, *DaT* dopamine transporter, *dPD* depressed PD, *HAM-D* Hamilton Depression Scale, *HARS* Hamilton anxiety rating scale, *HDRS* Hamilton depression rating scale, *MADRS* Montgomery–Asberg depression rating scale, *ndPD* non-depressed PD, *PD* Parkinson’s disease, *PET* positron emission tomography, *POMS* profile of mood state, *SCID* structured clinical interview for DSM-IV axis I disorders, *SHPS* Snaith–Hamilton pleasure scale, *SPECT* single-photon emission computed tomography, *SSRI* selective serotonin reuptake inhibitors, *STAI* state trait anxiety inventory, *UPDRSIII* unified Parkinson’s disease rating scale part III

- Anxiety

Anxiety composes a range of disorders, which can be classified into three categories (anxiety disorder, obsessive-compulsive disorder, and trauma and stressor-related disorder).^75^ Being a disorder, which can coexist with depression,^76^ the pathophysiology of anxiety is thought to be dopaminergic in part. Anxiety has also been shown to be a dopaminergic medication-related phenomenon evident in the dominant relationship with non-motor fluctuations in PD.^77^ Studies from Weintraub and colleagues, Erro and colleagues, and others (Table 2) have reported a reduction in dopaminergic uptake in the right striatum of anxious PD patients. However, when assessing different forms of anxiety, studies have not reported the same trend. Moriyama and colleagues, and Kaasinen and colleagues reported a positive correlation between dopaminergic DaT uptake within the striatum and social anxiety or personality traits and anxiety in patients, respectively.^78,79^ The variation found here, suggests anxiety to be heterogeneous in origin with a partial dopaminergic basis. Remy and colleagues demonstrated a negative correlation between the severity of anxiety with binding at the locus coeruleus and bilateral amygdala using ^11^C-RTI32, forging the concept of a DA-noradrenergic system involvement in PD-anxiety.^69^ This is supported by the understanding that both noradrenergic and dopaminergic pathways project from the locus coeruleus to sites including the amygdala and striatum.^80,81^

- Apathy

One third of PD patients experience apathy,^82^ characterized by a state of emotion with reduced motivation and a sense of reduced goal-directed behaviour, and ambition.^83^ Several types of apathy have been described and there is recognition that at least in part, apathy has a dopaminergic origin possibly through the involvement of the mesocorticolimbic circuit.^84–86^ Thobois and colleagues used [^11^C]-raclopride, a DA D2/D3 receptor ligand, and reported that PD-apathy patients had reduced synaptic DA release in the mesocorticolimbic system.^87^ In pure apathy (non-demented and non-depressed) PD patients, there has been a demonstration of reduced dopaminergic uptake in the striatum.^4,69^ However recently, Chung and colleagues have demonstrated there to be no association of striatal dopaminergic binding in early PD with apathy.^88^ Unfortunately, currently there is little evidence exploring the association of PD-apathy with dopaminergic dysfunction independent of other neuropsychiatric conditions. However, the reduced caudate uptake is mirrored in other neurodegenerative studies such as Dementia with Lewy bodies,^89^ Alzheimer’s disease,^90^ and frontotemporal dementia.^91^ The use of subthalamic nucleus deep brain stimulation (STN-DBS) in PD has itself been identified as being associated with inducing postoperative apathy.^92^ However, evidence exists showing there to be some predisposition to this risk factor in these patients including DA agonist withdrawal syndrome.^93–95^ Studies have explored mesolimbic dopaminergic dysfunction in STN-DBS induced apathy in PD patients^54,96,97^ finding there to be different mechanisms at play between early and late PD. Therefore, initially the dopaminergic mesocortical system is involved due to the relative sparing of the nigrostriatal dopaminergic system.

- Cognitive Impairment (CI)

CI and dementia have been associated with PD. Around 40% of patients have CI^98^ at early-stage of PD,^99^ and around 80% of patients may experience PD-related dementia (PDD) at a late-stage.^100,101^ Mild CI in PD (MCI) is also somewhat prevalent, where a recent review from the Movement Disorders Society (MDS) task force reports a prevalence of 27% (range 19–38%).^102^ A dopaminergic basis of cognitive impairment is possible and using ^18^F-DOPA PET, studies have demonstrated reduced dopaminergic uptake in PD patients at different stages of their condition with CI or PDD^99,103–109^ (Table 3), especially in the caudate nucleus.^110–112^ PDD is known to be a late-manifestation in PD.^113^ However, conversely some patients have been shown to present with early dopaminergic uptake changes within frontal structures critical to cognitive and executive function^99^; thus cognitive impairment can be an early-manifestation in PD. Three SPECT studies have shown there to be an association between reduced dopaminergic uptake and cognitive impairment in PD and CI as well as PD and MCI,^114–116^ however the authors do not use a uniform cognitive function test making their clinical definition of CI vary slightly. Nonetheless, they all report a significant correlation between striatal dopaminergic defect (more commonly unilateral and contralateral to the most affected side) and cognitive impairment existing in these PD patients (Table 3).

**Table 3:** Dopaminergic basis of NMSS Domain 3 (cognitive impairment) pathophysiology in PD

Author	Year	NMSs	Radiotracer	Demographics	Results	Analysis
Holthoff et al.^105^	1994	Cognitive impairment	^18^F-DOPA	7 pairs of twins discordant for PD underwent PET imaging.	Twin groups (PD and control) have significantly reduced ^18^F-DOPA uptake (*p* = <0.05). PD twins presented this reduction globally throughout the striatum. The control twins showed impaired ^18^F-DOPA uptake in at least one striatal region. Verbal memory processing was most impaired in PD twins (*p* = <0.05), however 6 co-twins also presented similarly significant impairment.	This is an important PET study, first to address genetic susceptibility and in vitro imaging in PD.
Marie et al.^112^	1999	Cognitive impairment	^11^C-S-NMF	10 non-demented, non-depressed PD patients underwent frontal executive tests, OA, CAL, and BPP.	A strongly significant correlation was found between right caudate binding and OA performance (*r* = −0.79, *p* = <0.02). Somewhat less significant, but converse correlations were observed between putamen binding and CAL performance (*r* = 0.71, 0 = <0.05; *r* = 0.64, *p* = <0.05; left and right putamen respectively). No such significant correlations were noted with BPP.	Data suggests caudate dopaminergic dysfunction may be the cause of PD-executive function impairment. This is another important early study in a small number of patients but its conclusions have been supported in succeeding studies.
Müller et al.^114^	2000	Cognitive impairment	^123^I-β-CIT	20 PD patients and 20 healthy controls underwent evaluation with MMSE, DS-F, DS-B, WMS-R, DOT, and RS.	Significant correlations between prefrontal task performance and β-CIT ratios for both the caudate head and putamen were seen (*p* = <0.05). Reading performance did not correlate however.	This is an early study which has been supported by later studies showing nigrostriatal dopaminergic dysfunction which correlates to the cognitive status in PD patients. The authors scanned and assessed patients in the “on” state but did not present any data on LEDD and whether there are correlations between LEDD and test scores. This could have influenced the results strongly since the authors claim that dopaminergic dysfunction may be the cause for executive function impairment.
Rinne et al.^111^	2000	Cognitive impairment	^18^F-DOPA	28 PD patients and 16 healthy controls underwent PET imaging alongside cognitive tests including MMSE and neuropsychological evaluation	There was reduced FDOPA uptake in the putamen (36% of control mean, *p* = <0.001), caudate (61% of control mean, *p* = <0.001) in PD patients, and frontal cortex in relation to neuropsychiatric tests in PD patients.	There may be dopaminergic dysfunction in cognitive impairment PD. One of the earliest controlled PET studies addressing cognitive and dopaminergic function in PD. The data has been subsequently replicated in many studies (see below).
Duchesne et al.^116^	2002	Cognitive impairment	^123^I-β-CIT	10 PD patients and 10 controls underwent a range of cognitive tests.	The simultaneous processing condition but not the selective or the competitive conditions took significantly more time for patients with PD-OFF than for either the control subjects or the patients with PD-ON. PD patients with PD-OFF took significantly more time than controls (*p* = <0.01) and PD-ON patients (*p* = <0.05) for the simultaneous processing condition only (not selective/ competitive conditions).	An older small controlled study, which has been replicated several times suggesting nigrostriatal dopaminergic dysfunction may be implicated in PD cognitive processing, according to the results.
Ito et al.^106^	2002	Cognitive impairment	^18^F-DOPA	10 non-demented PD patients, 10 PDD patients and 15 normal controls were recruited. Cognitive tests included MMSE.	PDD had a reduced ^18^F-DOPA uptake in bilateral striatum, midbrain and anterior cingulate area (*p* = <0.001). Relative differences in uptake were observed bilaterally in the caudate, anterior cingulate gyrus and ventral striatum between PD and PDD patients (*p* = <0.001).	The study suggests that PDD is associated with impaired mesolimbic and caudate function, although cognitive assessments could have been more detailed.
Brück et al.^99^	2005	Cognitive impairment	^18^F-DOPA	21 non-demented PD patients and 24 healthy controls underwent imaging and multiple cognitive tests including MMSE, CERAD, WAIS-R.	PD patients had, as was expected based on previous work, decreased striatal ^18^F-DOPA uptake compared to controls, however much of the cortex showed increased uptake. DLPFC ^18^F-DOPA uptake correlated with VIG reaction time (*p* = 0.013) and both the MFC and AC showed negative correlation with classic Stroop effect (*p* = 0.01). No significant correlations were found between cognitive testing and striatal uptake.	This is an important study showing increased cortical DaT uptake and a possible compensatory dopaminergic role in the brain network.
Cheesman et al.^107^	2005	Cognitive impairment	^18^F-DOPA	16 non-demented, non-depressed PD patients evaluated using TOL-SPT, VWMT.	Significant positive covariation was found between the right caudate and TOL score as determined by statistical paramertric mapping (*p* = 0.031). Similar covariation was seen between the left anterior putamen and performance in VWMT testing (*p* = 0.012).	A link between striatal dopaminergic defect and early executive function impairment in PD could be suggested on the basis of this study. But no control group was used. Surprisingly, PD motor patterns did not correlate with putamen DaT binding.
Cropley et al.^104^	2008	Cognitive impairment	^18^F-DOPA, ^11^C-NNC 112	15 non-demented non- depressed PD patients and 14 healthy controls. MMSE, DRS-2, WCST, and BDI were conducted.	No significant regional differences were observed between patients and controls with regards to D1-receptor density and in overall frontostriatal performance.	Analysis suggests that decreases in putaminal K_i_ predicted WCST performance in PD. This is an multimodal imaging study and as such, draws importance to advance in DA receptor basis of frontal cognition.
Jokinen et al.^103^	2009	Cognitive impairment	^18^F-DOPA	19 treated PD patients and 21 healthy controls took part with 12 undergoing cognitive tests including CERAD, WMS-R, WAIS-R, MMSE.	A positive correlation was found between the ^18^F-DOPA uptake of left ventral caudate and verbal memory (*r* = 0.72, *p* = 0.009), right ventral caudate and visual memory (*r* = 0.61, *p* = 0.037), and right ventral caudate and CERAD (*r* = 0.77, *p* = 0.003).	The analysis points towards reduced dopaminergic activity being able to impair cognitive performance tests. This is a powerful PET study with the use of controls.
Arnaldi et al.^109^	2012	Cognitive impairment	^123^I-FP-CIT	30 de novo, drug naïve PD patients underwent MMSE, ADL, GDS and other neuropsychiatric assessments.	Verbal memory and language task performance were significantly impaired in the posterior parieto-temporal region of the less affected side and was predicted by Dat uptake (*r* = 0.42, *p* = 0.0005). DaT caudate uptake in the less affected hemisphere combined with UPDRS-III score predicted decline in both executive (*r* = 0.54, *p* = 0.0001) and visuospatial (*r* = 0.56, *p* = 0.0001) function.	A dysfunctional dopaminergic basis is therefore proposed for some level of cognitive decline in PD. The strength of this study is the assessment in a reasonable drug naïve PD population supporting the role of dysfunctional dopaminergic basis and cognitive decline in PD.
Niethammer et al.^108^	2013	Cognitive impairment	^18^F-DOPA, ^123^I-FP-CIT	17 RH non-demented PD patients underwent imaging including PDCP.	The authors find a strong inverse correlation between PDCP scores and DaT binding in the caudate nucleus (*r* = −0.67, *p* = <0.005)) and putamen (*r* = −0.51, *p* = <0.05).	They therefore suggest there to be dopaminergic loss between caudate and the cognitive-network in PD. This is an interesting study but does not add any substantial new information.
Pellecchia et al.^115^	2015	Cognitive impairment	^123^I-FP-CIT	34 de novo, drug-naïve PD patients separated into those with (*n* = 15) and without (*n* = 19) MCI underwent neuropsychological battery.	DAT availability in average striatum, caudate, and putamen (more and less affected) was lower in MCI and in non-MCI patients with PD but not significantly different.	There’s some suggestions of striatal DA depletion contributing to cognitive defect in PD. This is an interesting study in drug naïve PD and addressing MCI vs. non-MCI PD. Supports a subtype concept and also a non-dopaminergic origin of MCI even in early PD.

^11^ *C-NNC 112* 8-chloro-7-hydroxy-3-methyl-5-(7-benzofuranyl)-2,3,4,5-tetrahydro-IH-3-benzazepine, ^11^ *C–S–NMF* ^11^C–S–Nomifensine, ^123^ *I-b-CIT* [(123)I]2beta-carbomethoxy-3-(4-iodophenyl)tropane, ^123^ *I-FP-CIT* [123I]-2β-carbomethoxy-3β-(4-iodophenyl)-N-(3-fluoropropyl) nortropane, ^18^ *F-DOPA* 18F-dihydroxyphenylalanine, *AC* anterior cingulate, *ADL* activities of daily living, *BDI* beck depression inventory, *BPP* brown Peterson paradigm, *CAL* conditional associative learning, *CERD* Consortium to Establish a registry for Alzheimer’s disease, *DaT* dopamine transporter, *DLPFC* dorsolateral prefrontal cortex, *DOT* digit ordering task, *DRS-2* dementia rating scale-2, *DS-B* digit span backwards, *DS-F* digit span forward, *GDS* geriatric depression scale, *MCI* mild cognitive impairment, *MFC* medial frontal cortex, *MMSE* mini mental state examinations, *OA* object alternation, *PD* Parkinson’s disease, *PDCP* PD cognition-related metabolic pattern, *PDD* Parkinson’s disease dementia, *PET* positron emission tomography, *RS* reading span, *SPECT* single-photon emission computed tomography, *TOL-SPT* tower of London spatial planning task, *VIG* sustained attention measure test, *VWMT* verbal working memory task, *WAIS-R* Wechsler Adult intelligence scale-revised, *WCST* Wisonsin card sorting test, *WMS-R* Wechsler memory scale-revised

The pathophysiology of cognitive impairment in PD may also involve the brainstem and corticostriatal pathway with cholinergic dysfunction.^117–121^ Using 2-^18^F-FA-85380 PET, studies have shown there to be cholinergic dysfunction of not just the striatum, but also the cerebellum, pons, and thalamus.^122,123^ In PDD, studies using N-^11^C-methyl-4-piperidyl-acetate (^11^C-PMP) acetylcholinesterase (AChE) PET have reported cholinergic degeneration.^124,125^ Bohnen and colleagues used ^11^C-PMP AChE PET on PDD patients finding a strong correlation of reduced radiotracer uptake with performances on working memory, attentional, and executive function tests suggesting a dominant cholinergic basis to these functions.^126,127^

### NMSS mood and apathy: summary statement

**Fig. 3:**
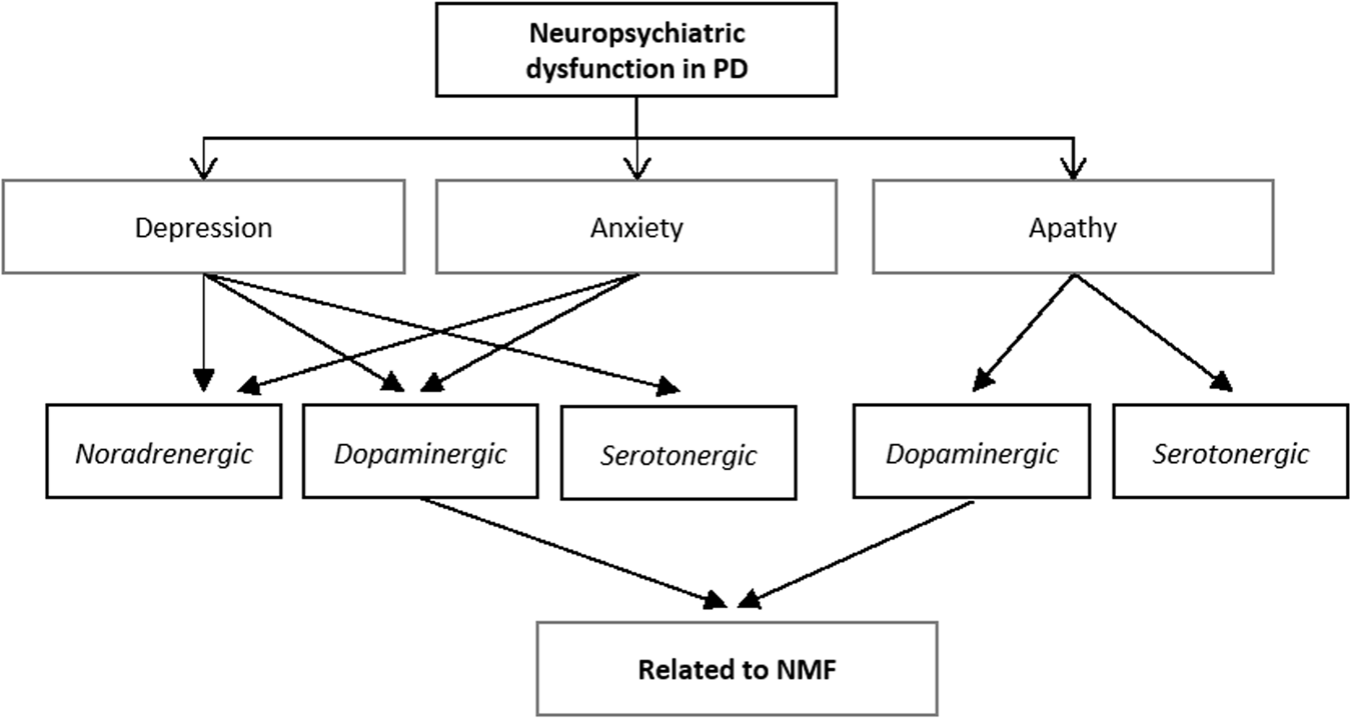
Summary of neuropsychiatric dysfunction in PD and the possible pathway’s involved in their pathophysiology. *PD* Parkinson’s disease, *NMF* non-motor fluctuations

Depression, apathy, and anxiety are often grouped together despite their heterogeneity in presentation and clearly apathy is a distinct NMS in its own right with several subtypes.^83^ Depression, anxiety, and aspects of apathy appear to have partial dopaminergic dysfunction, as per evidence from dopaminergic imaging (Fig. 3). The role of dopaminergic pathology in PD depression is far from clear and as such, in this review we have demonstrated the knowledge so far (see Table 3). Vast evidence is emerging to support serotonergic pathology as having clearer implications in PD depression,^7^ this is further supported by reduction in the midbrain FP-CIT SPECT DaT binding most likely reflecting serotonergic pathology rather than dopaminergic. Whilst serotonergic pathology may be at fault in PD depression, the spectrum of anxiety disorders may have noradrenergic, as well as dopaminergic involvement. By means of dopaminergic imaging, apathy has been demonstrated to have a mesocorticolimbic dysfunction. Nonetheless, the need to explore these non-dopaminergic bases is required to further understand the spectrum of conditions such as apathy, where specific PD research is lacking.

DaT imaging has shown supportive evidence for a dopaminergic dysfunction in PD cognitive impairment. With the complexity that cognition presents with and the level of neurotransmitters involved, an expectation of both dopaminergic and cholinergic dysfunction seems possible.

## NMSS domain 4: perceptional disorders

Perceptional disorders in PD, ranging from VH to delusions, are particularly prevalent with one-in-four PD patients experiencing VH.^128–132^ A dopaminergic basis of VH and other perceptional disorders is being researched. A cortico-striato-thalamocortical dysfunction has been suggested,^89,133,134^ however at present, work is required to determine and distinguish the differing forms of hallucinations. Recent data provides evidence of a dopaminergic basis in VH^135^ (Table 4), but not auditory hallucinations.^136^ Several studies also support non-dopaminergic involvement through both serotonergic^10^ and cholinergic^129,130,137–140^ means.

**Table 4:** Dopaminergic basis of NMSS Domain 4 (perception disorders) pathophysiology in PD

Author	Year	NMS	Radiotracer	Demographics	Results	Analysis
Kiferle et al.^135^	2014	VH	^123^I-FP-CIT	18 non-demented PD patients with VH and 18 non-demented PD patients without VH.	Significant reduction in baseline right caudate uptake (*p* < 0.05) in patients with VH. With regards to putamen and contralateral caudate uptake, no significant differences were observed between groups.	Not a particularly notable study as the groups studied are difficult to define and role of medication induced hallucinations complicates the findings. The study lacks baseline neuropsychological evaluation, which would have been useful.

^123^ *I-FP-CIT* [123I]-2β-carbomethoxy-3β-(4-iodophenyl)-N-(3-fluoropropyl) nortropane, *PD* Parkinson’s disease, *VH* visual hallucinations

Lower DaT binding in the striatum in early PD measured by [^123^I] [ß]-CIT tracer (DaTscan) is associated with increased prospective risk of psychosis spectrum at 5 years.^134^ It is unclear whether this binding reduction is the underlying mechanism of the psychosis spectrum or, whether an indirect association, for example reflecting more extensive neurodegenerative involvement in the psychosis spectrum, is present. A serotonergic imaging study using the 5HT_2A_ receptor ligand setoperone-F^18^ identified increased binding in patients with VH in ventral occipito-temporal regions and bilateral frontal cortex.^10^ In contrast, a 5HT_1A_ receptor binding study in post-mortem tissue found no association with psychosis spectrum, although 5HT_1A_ binding was elevated in PD irrespective of hallucination status in sublayers of orbito-frontal, ventral temporal, and motor cortex.^141^

### NMSS perceptional disorders: summary statement

Currently, DaT imaging has not supported perceptional disorders as having a dopaminergic basis. Rather, recent studies suggest a multifactorial origin of hallucinations including alterations in dopaminergic, serotonergic, and cholinergic systems. Further longitudinal imaging studies involving the aforementioned neurotransmitters and pathways are required.

## NMSS domain 5: attention defects

PD patients have been noted to experience various attentional function deficits, including visuospatial^142^ and during performance of tasks requiring a switch of behaviour.^143,144^ The evidence thus far supports a dopaminergic pathology underlying attention deficits in PD. Work from Rinne and colleagues (Table 5) find correlation between reduced ^18^F-DOPA uptake in the caudate and frontal cortex with attentional and working memory deficit. Further evidence supports dopaminergic deficit within the frontal cortex (more specifically the medial portion), alongside the anterior cingulate and the dorsolateral prefrontal cortex.^99^ However, Bohnen and collegues showed a robust correlation with cortical AChE activity with attention and working memory; suggestive of cholinergic involvement.^124^

**Table 5:** Dopaminergic basis of NMSS Domain 5 (attention and memory) pathophysiology in Parkinson’s disease

Author	Year	NMS	Radiotracer	Demographics	Results	Analysis
Rinne et al.^111^	2000	Attention	^18^F-DOPA	28 PD patients and 16 healthy controls were assessed via MMSE, detailed neuropsychological assessment including tests for frontal lobe function.	Reduction of ^18^F-DOPA uptake in the caudate and frontal cortex is associated with a poor performance in tests requiring working memory and attention (*p* = 0.001).	This controlled early PET study highlights the possible dopaminergic basis of working memory and attention.
Brück et al.^99^	2005	Attention	^18^F-DOPA	21 non-medicated patients, non-demented PD patients and 24 healthy controls.	Increased tracer uptake in the medial frontal cortex and anterior cingulate correlated negatively with reaction time requiring suppressed attention (*p* = 0.01). Increased uptake in dorsolateral prefrontal cortex showed a positive correlation with sustained attention (*p* = 0.014).	This is an important controlled PET study showing a possible role of compensatory cortical mechanisms at play. The study did have a large interval between neuropsychological testing and imaging (66 days on average) which may be need to be shorter in a subsequent study.

^*18*^ *F-DOPA* 18F-dihydroxyphenylalanine, *MMSE* mini mental state examination, *PD* parkinson’s disease

### NMSS attention defects: summary statement

Attention defects in PD are likely to be mediated through cholinergic dysfunction although a dopaminergic pathophysiology is also suggested by dopaminergic imaging studies. This is supported by the concept of frontal lobe and basal ganglia disturbances, which over the course of time may progressively worsen.

## NMSS domain 6: gastrointestinal tract

Gastric dysfunction in PD is a prevalent issue with symptoms ranging from drooling, dysphagia, and constipation to gastroparesis^145,146^ with constipation being suggested as a pre-motor marker in a recent Danish study.^147^ There is currently no evidence that supports a dominant dopaminergic pathogenesis for gastric symptoms, whereas cholinergic dysfunction has been suggested by Gjerloff and colleagues who investigated the parasympathetic involvement of AChE binding using ^11^C-donepezil PET. They report a significant decrease in ^11^C-donepezil uptake in the small intestines and pancreas, proposing cholinergic dysfunction of the enteric nervous system in PD.^148^

### NMSS gastrointestinal tract: summary statement

Dopaminergic imaging studies supporting a dopaminergic basis to the pathophysiology of gastric dysfunction in PD are currently not explored. The use of ^11^C-donepezil PET however has suggested there to be an early enteric cholinergic dysfunction in PD.

## NMSS domain 7: urinary dysfunction

Referred to collectively as lower urinary tract symptoms (LUTS), one of the key and most frequent autonomic problems in PD is bladder dysfunction.^149^ PD patients experience elevated urinary frequency, urgency, nocturia, incontinence, and voiding.^150^ There is evidence for an underlying dopaminergic basis in the pathophysiology of LUTS (Table 6). Dopaminergic influences in the micturition reflex are present in inhibitory pathways arising from dopaminergic Substantia Nigra pars Compacta fibres, whilst the stimulatory affect arises from dopaminergic ventral tegmental area (VTA) fibres.^151,152^ Work from Winge and colleagues further supports a dopaminergic dysfunction (Table 6).^201^ However, the micturition reflex and LUTS are controlled by a number of neurotransmitters including DA, serotonin, NA, and ACh.^153^ Moreover, PD-LUTS patients do not necessarily respond to levodopa or dopaminergic treatment but may instead require anticholinergic treatment suggesting a dominant underlying cholinergic basis.^152,154^

**Table 6:** Dopaminergic basis of NMSS Domain 6 (urinary dysfunction) pathophysiology in PD

Author	Year	NMS	Radiotracer	Demographics	Results	Analysis
Sakakibara et al.^150^	2001	Urinary	^123^I-β-CIT	11 PD patients with LDOPA treatment.	Reduction in nigrostriatal dopaminergic function, notably in the caudate (*p* = 0.01, right side; 0.05, left side), anterior and posterior putamen (*p* = 0.05, both right side) of the group of patients with urinary dysfunction.	This is one of the few studies addressing a key non-motor symptom, urinary dysfunction. However, the finding is non-specific and do not suggest a strong pathophysiological basis. Furthermore, the urinary dysfunction symptoms were not represented well in the small sample and no urodynamic evaluation was obtained.
Winge et al.^201^	2005	Urinary	^123^I-FP-CIT	18 PD patients underwent imaging.	Patients with bladder symptoms had reduced uptake in the putamen and caudate (*p* = 0.03) with correlation in caudate degeneration and symptom severity.	The study suggests a dopaminergic basis for LUTS. Another study of urinary dysfunction with non-specific findings. The relationship with caudate is interesting and warrants further exploration. Lack of controls and the arbitrary cut off in the urinary questionnaire, limits the validity.

^*123*^ *I-b-CIT* [(123)I]2beta-carbomethoxy-3-(4-iodophenyl)tropane, ^*123*^ *I-FP-CIT* [123I]-2β-carbomethoxy-3β-(4-iodophenyl)-N-(3-fluoropropyl) nortropane, *LUTS* lower utinary tract symptoms*, PD* parkinson’s disease

### NMSS urinary dysfunction: summary statement

There is evidence for a dopaminergic association with urinary dysfunction in PD patients particularly D1 receptor activity.^150^ However, urinary dysfunction in PD is likely to have a mixture of dopaminergic and cholinergic mechanisms.

## NMSS domain 8: sexual dysfunction

Sexual dysfunction is a common problem for many PD patients^155,156^ and erectile dysfunction (ED), hyper-sexuality, loss of lubrication, loss of libido, and involuntary urination during sex are just some such symptoms.^149,157–161^ There is poor evidence that dopaminergic dysfunction may underlie sexual dysfunction. ED can be caused by both vascular and hormonal, as well as neurological pathologies^162^ and using PET and fMRI studies, there has been identification of dopaminergic and serotonergic structures, such as the insula, caudate nucleus, putamen, thalamus, and nucleus accumbens as likely being involved in ED pathogenesis.^163–167^ However, sexual issues in PD could also be drug induced manifestations of impulse control disorders (ICD).^168–172^ Discussion of functional imaging based studies of ICD is beyond the scope of this review.

### NMSS sexual dysfunction: Summary statement

Symptoms of sexual dysfunction vary in PD, and ED may be in part driven by dopaminergic mechanisms although there are no specific dopaminergic imaging studies.

## NMSS domain 9: miscellaneous

- Olfactory changes

Braak and colleagues proposed the idea that PD-pathology begins in extra-nigral structures, hence why olfactory dysfunction is a common initial prodromal symptom for many PD patients.^28^ There is some evidence that dopaminergic dysfunction is responsible for olfactory symptoms (Table 7). Using SPECT imaging, several studies now have found there to be supporting evidence of DaT uptake reduction in hyposmic patients.^173–180^ Scherfler and colleagues offer concordant evidence that both nigral and olfactory tract degeneration parallels that of putaminal dopaminergic dysfunction in PD patients,^202^ although not all studies agree.^181–183^ In contrast, two studies present evidence that DA agonists are ineffective in treating hyposmic symptoms however, this may be because the damage is simply too excessive.^184,185^

**Table 7:** Dopaminergic basis of NMSS Domain 9 (miscellaneous) pathophysiology in PD

Author	Year	NMS	Radiotracer	Demographics	Results	Analysis
Bohnen et al.^175^	2007	Olfactory	^11^C-β-CIT	27 PD patients and 27 healthy controls underwent UPSIT testing.	The authors present evidence of significant correlations between dorsal striatal DaT excitation and total UPSIT (R(S) = 0.44, *p* = 0.023) scores.	Therefore, PD-hyposmia may have dopaminergic basis to its pathophysiology. This is an important controlled study addressing olfaction and a possible dopaminergic basis. The study does however have variation in their patients which were not accounted for, such as some being drug naïve, some newly diagnosed, and other on several mediations.
Berendse et al.^178^	2011	Olfactory	^123^I-FP-CIT	96 PD patients underwent UPSIT	Olfactory deficit in PD correlated with striatal DaT binding in the most affected putamen and caudate nucleus (*p* = 0.03), and least affected putamen and caudate nucleus (*p* = 0.01).	This is a large uncontrolled study, adding to the observations of Bohnen et al 2007, suggesting that dopaminergic dysfunction occurs in early hyposmic PD pathogenesis. The study sample had differences in treatment which were not reported, nor were results analysed with treatment as independent variables, which may provide interesting results.
Lee et al.^209^	2016	Weight	^18^F-DOPA	398 PD patients underwent imaging, BMI measurements	All sub regions of the striatum demonstrated a significant positive correlation with BMI as follows: anterior putamen (*r* = 0.159, *p* = 0.001), posterior putamen (*r* = 0.126, *p* = 0.012), ventral striatum (*r* = 0.136, *p* = 0.007), caudate nucleus (*r* = 0.15, *p* = 0.003), and total striatum (*r* = 0.161, *p* = 0.001).	This study suggests that low BMI may correlate with dopaminergic dysfunction in PD. Patients with BMI less than 18.5 had even lower striatal DaT activity, suggesting effects of undernourishment on dopaminergic function. This is an important and thus far a unique study with a very large sample size addressing body weight and PD. Altered body weight now thought to be a possible predictor of dyskinesia’s as well as prognostic marker.

^*123*^ *I-b-CIT* [(123)I]2beta-carbomethoxy-3-(4-iodophenyl)tropane, ^*123*^ *I-FP-CIT* [123I]-2β-carbomethoxy-3β-(4-iodophenyl)-N-(3-fluoropropyl) nortropane, ^*18*^ *F-DOPA* 18F-dihydroxyphenylalanine, *BMI* body mass index, *DaT* dopamine transporter, *PD* parkinson’s disease, *UPSIT* smell identification test

- Weight changes

PD patients characteristically undergo diet/metabolism-unassociated weight loss starting early in the course of the disease.^186,187^ Based on available evidence, a dopaminergic basis for weight change in PD patients is not unexpected, however only one study by Lee and colleagues explores this (Table 7). DA is involved in modulating the reward and motivational properties of food intake^188^ causing problems in weight gain and loss.^189^ Weight gain is commonly associated with DA agonist treatment due to the side effects of ICD, specifically compulsive binge eating.^190–193^ However, evidence suggests a prominent serotonergic involvement, and weak potential for noradrenergic action^194^ as serotonin is thought to play a crucial role in modulation of appetite.^195^ In a study using ^11^C-DASB, a marker for SERT, Politis and colleagues demonstrated increased tracer binding in the rostral raphe nuclei, hypothalamus, caudate nucleus, and ventral striatum in PD patients with abnormal BMI changes.^9^ Interestingly, gain in BMI was associated with raised ^11^C-DASB binding in the anterior cingulate cortex when compared to those with reduced BMI. These findings imply that decreased levels of serotonin, due to elevated clearance, could lead to abnormal BMI changes. Furthermore, Sharma and colleagues propose introduction of a Park-weight subtype following observation, using standardized olfactory assessments, reporting PD patients with severe olfactory dysfunction correlate with having an increased risk of weight loss.^196,197^

### NMSS miscellaneous: summary statement

The pathophysiology of olfactory dysfunction in PD may have a dopaminergic basis, and in part clinical data supports this observation.^198^ However, involvement of the cholinergic system is also likely given the recent definition of the olfactory-limbic pathway.^199,200^ Abnormal weight change is a common symptom in PD, affecting patients early. Recent imaging studies suggest that alterations in dopaminergic as well as serotonergic systems give rise to pathological changes in weight. Areas identified to be involved comprise the striatum for dopaminergic changes and rostral raphe nuclei as well as hypothalamus for serotonergic alterations.

## Conclusion

To our knowledge, this is the first review that has summarised available evidence exploring the possible dopaminergic basis of NMS pathophysiology using the domains integral to the NMSS, a widely used validated measure of holistic NMS assessment. Hence, it also addresses an unmet need in this regard.

We have found there to be 12 NMS with imaging based evidence for at least in part, a dopaminergic pathophysiological basis (Table 8). The use of radiotracers has certainly evolved and as such tracers such as, ^99m^Tc-TRODAT-1 SPECT is not regularly used in research now due to its low unreliability and low specificity in comparison to other imaging modalities. Furthermore, we have highlighted key NMS which have non-dopaminergic pathophysiologic involvement (Table 9).

**Table 8:** Radiotracers used to assess dopaminergic NMS pathophysiology in PD

NMS/radiotracers	^123^I-IBZM	^123^I-IPT	^123^I-FP-CIT	^123^I-β-CIT	^18^F-DOPA	^99m^Tc-TRODAT-1	^11^C-RTI-32	^11^C-S-NMF	^18^F-FP-CIT
RBD	x	x	x						
RLS/PLM			x						
Fatigue				x	x				
Depression			x		x	x	x		
Anxiety			x		x	x	x		
Apathy			x				x		x
Cognition			x	x	x	x		x	x
Perception			x						
Attention					x				
Weight					x				
Bladder			x	x					
Olfactory			x	x

**Table 9:** Radiotracers available in investigating dopaminergic pathophysiology in NMS-PD

DaT	Vesicle transporter	Dopamine D2/D3 receptors
^123^I-FP-CIT	^11^C-DTBZ	^11^C-Raclopride
^123^I-β-CIT	^18^F-DTBZ	^11^C-FLB456
^123^I-altropane		^11^C-PHN0
^11^C-(MP)	**DA storage**	^18^F-fallypride
^11^C-CFT	^18^F-DOPA	^123^I-IBZM
^18^F-CFT		
^11^C-PE2I		
^18^F-FP-PE2I		
^99M^Tc-TRODAT-1		
^11^C-RTI32		

Adapted from Politis *et al.* ^201^

Our review findings are summarised in Table 10, where we have classified evidence for the neurotransmitters involved in the pathophysiology of NMS into four arbitrary categories (strong, moderate, weak, and conflicting evidence). We have catogorised stronger evidence as having open-label trials, or more than 3 publications demonstrating neuroimaging based evidence. Moderate evidence; as having clinical trials, or 2–3 studies presenting neuroimaging based evidence, while weak evidence is defined as having some clinical cases, or 1 study reporting neuroimaging based evidence. Finally, conflicting evidence is defined having 2 or more conflicting studies. This table presents the first summary of NMS in relation to the potential neurotransmitters involved in their pathology. It also shows how some NMS such as insomnia, anhedonia, and delusions have had no or very little exploratory research conducted.

**Table 10:** Neurotransmitters involvement in the NMS of PD pathophysiology

Domain	Description	DA	5HT	NA	ACh
1	Cardiovascular dysfunction				
	Orthostatic hypotension			+++	
	Black-out			++	
2	Sleep/Fatigue				
	EDS	++	++		
	Fatigue	+/-	++		
	Insomnia				
	REM behaviour disorder (RBD)	++			++
	RLS and periodic limb movements	+++	++		
3	Mood/ Apathy				
	Anhedonia				
	Apathy	++			
	Anxiety	+++	+	++	
	Depressed	+	++	+	
4	Perceptual problems				
	Hallucinations	+	+++		+
	Delusions				
	Double vision	++	++		
5	Attention/memory				
	Attention deficit	+++			++
	Memory deficit/ cognitive impairment	++			+++
	Confusion	+			
6	Gastrointestinal tract				
	Dribbling	+			
	Dysphagia	+			++
	Constipation	+			++
7	Urinary				
	Urgency	+++			
	Frequency	+++			
	Nocturia	+++			
8	Sexual dysfunction				
	Loss of libido	+			
	Erectile dysfunction	+			
9	Miscellaneous				
	PD-related pain	+			
	Olfactory dysfunction	+++			
	Weight change	+++	++		
	Excessive sweating	+			

+++: Strong evidence from clinical studies, 3 or more neuroimaging evidence

++: Moderate evidence from clinical studies, 2–3 neuroimaging evidence

+: Weak evidence from single case reports, or 1 neuroimaging evidence

+/−: Conflicting evidence from 2 or more studies

*DA* dopamine, *5HT* serotonin, *NA* noradrenergic, *ACh* acetylcholine, *REM* rapid eye movement
