# Author Correction: Presynaptic dopaminergic terminal imaging and non-motor symptoms assessment of Parkinson’s disease: evidence for dopaminergic basis?

**DOI:** 10.1038/s41531-023-00464-6

**Published:** 2023-02-03

**Authors:** M. A. Qamar, A. Sauerbier, M. Politis, H. Carr, P. A. Loehrer, K. Ray Chaudhuri

**Affiliations:** 1 National Parkinson’s Foundation International Center of Excellence, King’s College London and King’s College Hospital NHS Foundation Trust, London, UK; 2 Neurodegeneration Imaging Group, Department of Basic and Clinical Neuroscience, Institute of Psychiatry, Psychology and Neuroscience (IoPPN), King’s College London, London, UK; 3 Department of Neurology, University Hospital Cologne, Cologne, Germany

Correction to: *npj Parkinson’s Disease* 10.1038/s41531-016-0006-9, published online 25 January 2017

In this article the author name PA Loehrer was incorrectly written as P Loehrer. The original article has been corrected.

